# Effect of Different References on Auditory-Evoked Potentials in Children with Cochlear Implants

**DOI:** 10.3389/fnins.2017.00670

**Published:** 2017-12-04

**Authors:** Maojin Liang, Jiahao Liu, Junpeng Zhang, Junbo Wang, Yuebo Chen, Yuexin Cai, Ling Chen, Yiqing Zheng

**Affiliations:** ^1^Department of Otolaryngology, Sun Yat-Sen Memorial Hospital, Sun Yat-Sen University, Guangzhou, China; ^2^Department of Otolaryngology, Sun Yat-Sen Memorial Hospital, Institute of Hearing and Speech-Language Science, Sun Yat-Sen University, Guangzhou, China; ^3^Department of Hearing and Speech Science, Xin Hua College of Sun Yat-Sen University, Guangzhou, China; ^4^Department of Medical Information and Engineering, Sichuan University, Chengdu, China; ^5^Department of Clinical Medicine, Sun Yat-Sen University, Guangzhou, China

**Keywords:** cochlear implant, nose reference, mastoid reference, montage average reference, reference electrode standardization technique, event related potential

## Abstract

**Background:** Nose reference (NR), mastoid reference (MR), and montage average reference (MAR) are usually used in auditory event-related potential (AEP) studies with a recently developed reference electrode standardization technique (REST), which may reduce the reference effect. For children with cochlear implants (CIs), auditory deprivation may hinder normal development of the auditory cortex, and the reference effect may be different between CIs and a normal developing group.

**Methods:** Thirteen right-side-CI children were recruited, comprising 7 males and 6 females, ages 2–5 years, with CI usage of ~1 year. Eleven sex- and age-matched healthy children were recruited for normal controls; 1,000 Hz pure tone evoked AEPs were recorded, and the data were re-referenced to NR, left mastoid reference (LMR, which is the opposite side of the implanted cochlear), MAR, and REST. CI artifact and P1–N1 complex (latency, amplitudes) at Fz were analyzed.

**Results:** Confirmed P1–N1 complex could be found in Fz using NR, LMR, MAR, and REST with a 128-electrode scalp. P1 amplitude was larger using LMR than MAR and NR, while no statistically significant difference was found between NR and MAR in the CI group; REST had no significant difference with the three other references. In the control group, no statistically significant difference was found with different references. Group difference of P1 amplitude could be found when using MR, MAR, and REST. For P1 latency, no significant difference among the four references was shown, whether in the CI or control group. Group difference in P1 latency could be found in MR and MAR. N1 amplitude in LMR was significantly lower than NR and MAR in the control group. LMR, MAR, and REST could distinguish the difference in the N1 amplitude between the CI and control group. Contralateral MR or MAR was found to be better in differentiating CI children versus controls. No group difference was found for the artifact component.

**Conclusions:** Different references for AEP studies do not affect the CI artifact. In addition, contralateral MR is preferable for P1–N1 component studies involving CI children, as well as methodology-like studies.

## Introduction

Event-related potentials (ERPs), with excellent temporal resolution, are one of the most informative and noninvasive methods of monitoring and studying the cognitive processes in the living brain. ERPs are linked in time with a physical or mental event and are typically extracted from scalp-recorded electroencephalogram (EEG) by means of signal averaging (Duncan et al., 2009).

In the auditory field, the latency and morphology of auditory evoked potentials (AEPs) can provide information about the maturation of the auditory system. There are several studies that reported that compared to children, adults show smaller amplitudes and latency in the P1 component (Ponton et al., 2000; Wunderlich and Cone-Wesson, 2006; Wunderlich et al., 2006; Shafer et al., 2015). P1 is described as a result of synaptic activity in the primary auditory cortex, thalamo-cortical projections, and intercortical recurrent activity (Ponton et al., 2000; Eggermont and Ponton, 2003). The latency of P1, as well as N1, AEPs decrease with age systematically in normal hearing children (Ponton et al., 2002). In cochlear implanted children, AEPs are also used to study auditory system plasticity and rehabilitation efficacy after regaining auditory information (Kral and Sharma, 2012; Sharma et al., 2015a). It is reported that compared to age-matched normal hearing children, CI individuals have larger P1 amplitudes and longer P1 latency (Kral and Sharma, 2012; Sharma et al., 2015a).

EEG is measured against a specific reference electrode. The reference electrode is the electrode keeping a relatively steady potential in ERP studies. The underlying assumption is that the reference should be electrically quiet; however, there is no such point on the human body surface (Yao, 2001; Nunez and Srinivasan, 2006). Fluctuation of the voltage at the reference electrode will lead to changes of the potential at the active electrode, even if the voltage at that point is actually stable. Thus, with different references, the voltage waveforms extracted from the same measuring electrode often show different results. Therefore, the choice of reference is a critical issue for obtaining reliable ERPs when investigating cognitive processing.

To minimize the possible effect of different references in ERP studies, different reference sites have been used, as lab personnel have historically used them, or as is widely used in most research, which we found from literature in this field (Wolpaw and Wood, 1982). The average reference is widely considered to be superior to all other known reference schemes because it is independent of any particular recording sites included in the EEG montage (Kayser and Tenke, 2015).

In cochlear implant (CI) users, CI stimulation creates electrical artifacts on the scalp that corrupt the EEG signal, which interfere with identification of the ERP components. The strength, morphology, and spatial distribution of the CI artifact are influenced by the type and location of the CI devices and the mode of stimulation. For example, devices running with bipolar electrodes in the CI show smaller artifacts on the scalp compared to the now commonly used monopolar-coupled electrodes (Gilley et al., 2006). Thus, the reference electrode location chosen may be of great importance.

Although there are several studies that showed that the AEP component, P1, can reflect the auditory cortex ability in processing auditory information (Wunderlich et al., 2006; Sharma et al., 2015b), unfortunately, we have not found sufficient recent articles discussing which reference is the most suitable one in an auditory P1 study. With respect to the reference site, our study aimed at comparing three commonly used references [nose reference (NR), mastoid reference (MR), and montage average reference (MAR)] and one technique, reference electrode standardization technique (REST) (Yao, 2001), to determine whether different references impact the AEP characteristics in CIs. A secondary purpose of this study was to evaluate which reference is preferable for AEP studies in CIs.

## Methods

### Participants

Thirteen patients (aged 4.37 ± 0.73 years) who had undergone surgical implantation of a multichannel CI device on their right side were recruited after ~1 year of cochlear device usage. In these patients who were diagnosed with congenital bilateral profound sensorineural deafness, the average age of cochlear implantation was 1.21 ± 0.09 years. Table 1 shows the demographic profiles of the CI participants. The etiology of deafness was unclear in all participants. None of the participants had any record of neurological or psychiatric illnesses. In addition, no inner ear or auditory nerve malformation was found during pre-operative CT and MRI evaluations. The peripheral hearing investigations revealed pure tone thresholds to 500, 1,000, 2,000, and 4,000 Hz stimuli in the 30–40 dB range in all participants. After surgery, all of the participants received standard speech rehabilitation from speech rehabilitation centers. Eleven children (aged 4.58 ± 0.52 years, matched with the age of cochlear implanted children) with congenital left external and middle ear malformation but normal hearing in the right ear were put in the control group. Ethical approval was obtained from the Institutional Review Board at Sun Yat-sen Memorial Hospital of Sun Yat-sen University before the study began. Written consent was obtained from the parents of all participants before any of the study procedures were conducted.

**Table 1:** Demographic characteristics of CI children.

**Participant code (gender)**	**Age at experiment (years)**	**Implant device**	**Age at implantation (years)**	**Duration of CI experiment (years)**
CI1 (M)	4.45	MEDEL SONATAti100	3.39	1.06
CI2 (M)	3.25	MEDEL SONATAti100	2.05	1.20
CI3 (F)	5.3	MEDEL SONATAti100	4.05	1.25
CI4 (M)	5.23	AB	4.15	1.08
CI5 (F)	2.79	COCHLEAR	1.52	1.27
CI6 (M)	4.6	COCHLEAR CI24RE	3.40	1.20
CI7 (F)	4.5	MEDEL SONATAti100	3.07	1.43
CI8 (F)	4.34	COCHLEAR CI24RE	3.19	1.15
CI9 (M)	5.09	MEDEL SONATAti100	3.90	1.19
CI10 (F)	4.22	MEDEL SONATAti100	2.93	1.29
CI11 (F)	4.79	AB	3.59	1.20
CI12 (M)	3.94	MEDEL SONATAti100	2.76	1.18
CI13 (M)	4.34	MEDEL SONATAti100	3.13	1.21

*Participants in the cochlear group all had undergone surgical implantation of a multichannel cochlear implant device on their right side*.

### AEP measurement

Participants were comfortably seated in front of a high-resolution VGA computer monitor at a viewing distance of ~1 m in a soundproof and electromagnetically shielded room. The participants watched silent movies throughout the entire experiment. Parents and participants were asked to avoid/minimize body movements. A DELL computer running the E-prime®2.0 program-generated 1,000 Hz pure tone stimulus elicited the AEPs. The pure tone was 60 ms in duration (5 ms rising and 5 ms descending) and was followed by inter-stimulus intervals (ISI) ranging from 600 to 800 ms. A total of 100 stimuli were delivered through loudspeakers in the booth, placed at a 45° angle on either side of the participants, ~1 m from the participants (75 dB SPL).

### EEG recording and analysis

A 128-channel electroencephalography (EEG) electrode recording system (Electrical Geodesics, Inc.) physically referenced to the vertex was used to record the AEPs. CI children with the external coil protected used plastic wrap during testing. The sampling rate for the EEG recording was 1 kHz, and all electrode impedances remained below 40 kΩ (Liang et al., 2014). The EEG recordings of each child were bandpass filtered offline at 0.1–30 Hz and segmented with 100 ms pre-stimulus and 600 ms post-stimulus time. Artifact rejection set at 200 μV was applied to EEG, and epochs were rejected if they contained any eye blinking (eye channel exceeded 140 μV) or eye movement (eye channel exceeded 55 μV). Bad channels were removed from the recording. The response waveforms evoked by the stimuli were obtained by averaging all valid segments. To test the effects of reference electrode difference, the original CZ-referenced EEG signals were re-referenced offline to (1) nose reference (NR), (2) left mastoid reference (LMR), (3) montage average reference (MAR), and (4) REST (Wolpaw and Wood, 1982), which was transformed from MAR. Reference-free or reference-independent potential could not be measured, which is why such all kinds of reference schemes were used in various research groups and institutes. Among all of the reference schemes, REST could reduce the effect of the reference and could improve analysis of temporal characteristics of ERP for some cases. The data were finally baseline corrected to the pre-stimulus period of −100 to 0 ms. The artifact rejection was conducted by the EGI program automatically.

CI artifact and P1–N1 complex (latency, amplitude) at Fz electrode for individual participants were analyzed. The highest positive amplitude between 90 and 180 ms was selected as P1. The N1 component was defined as the highest negative amplitude between 110 and 320 ms. In addition, the artifact was observed as the highest negative amplitude between 0 and 80 ms. Amplitudes of the P1, N1, and artifact peaks were measured from baseline to the peak value. Latencies were chosen at the highest amplitude of the peak.

## Results

### Data and explanation

The reference, whose AEP result of P1 could better distinguish the difference between the CI and control group, would be considered as a more preferable one in our study. The reason behind our consideration is that P1 is the biomarker of assessing cortical maturation in pediatric hearing loss (Liang et al., 2014); thus, the CI group would show differences with the control group on the P1 component, which is supported by the main effect of group in our study. In addition, the simple effect test result of the ideal reference should be consistent with it.

We also performed similar experiments in other components, such as N1 and artifact. Since researchers cannot reach an agreement to change these components in their study, we used them in second place of our study.

To compare results of different groups on the level of the reference, and only if the significant main effect of the group exists, we would perform a simple effect test whether the reference^*^ group interaction was significant or not.

**Figure 1:**
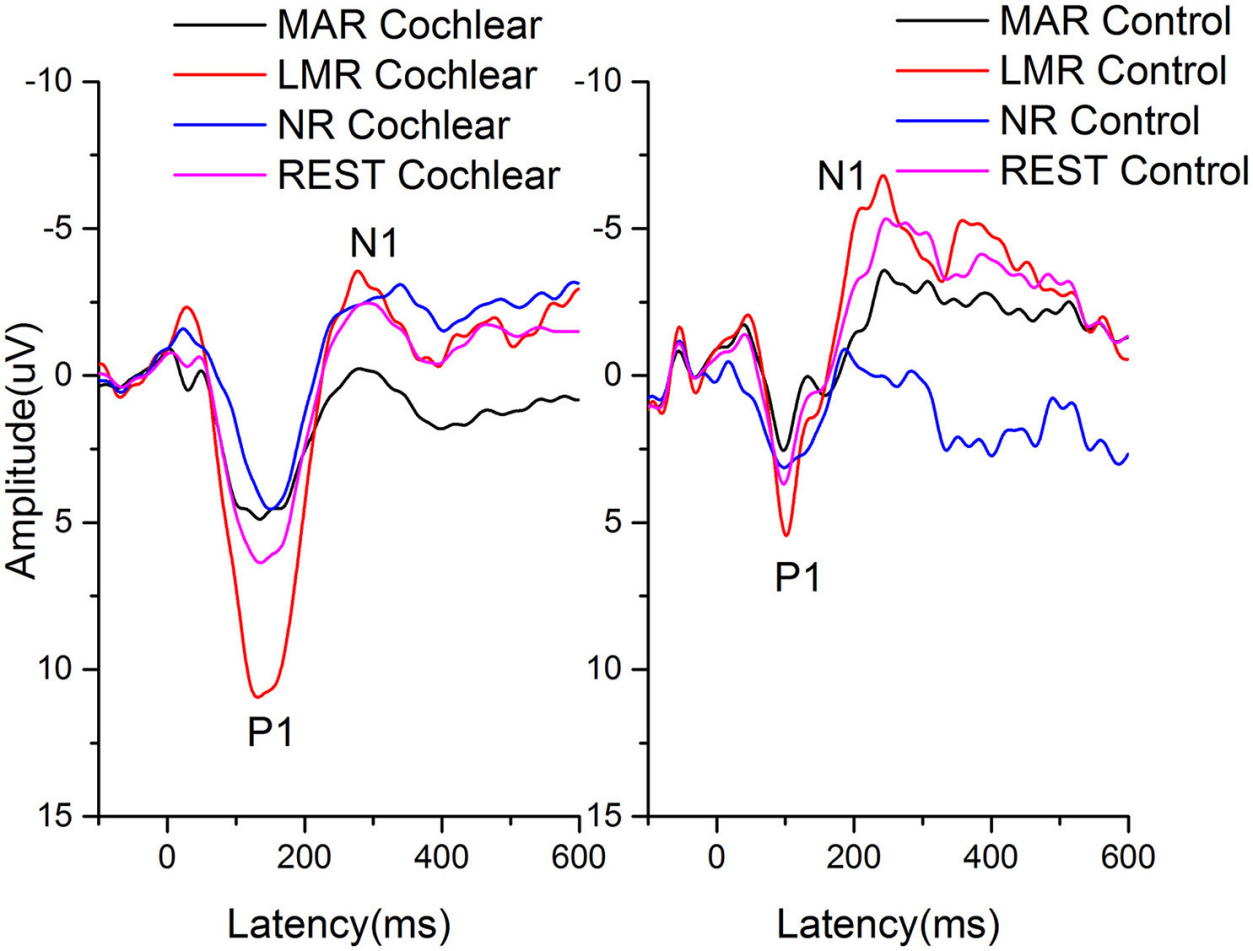
The grand average AEPs re-referenced offline to NR, LMR, MAR, and REST of the cochlear and control group at the vertex (Fz) electrode site are shown. Amplitudes are in microvolts on the vertical axis and time, in ms, is on the horizontal axis.

The grand average AEPs re-referenced offline to NR, LMR, MAR, and REST of the cochlear and control group at the vertex (Fz) electrode site are shown in Figure 1.

The latencies and amplitudes of P1, N1, and artifact recorded at the Fz electrode re-referenced offline to NR, LMR, and MAR, respectively, in the cochlear and control group are presented in Table 2. The latencies and amplitudes of the P1, N1, and artifact components were analyzed by two-way repeated-measures analysis of variance (ANOVA) with one between-group factor (group) and one within-group factor (NR, LMR, and MAR reference). The results of the simple effect test are shown in Tables 3, 4.

**Table 2:** P1, N1, and artifact latencies and amplitudes recorded at the Fz location.

		**Amplitude (ms)**			**Latency (uv)**		
		**P1**	**N1**	**Artifact**	**P1**	**N1**	**Artifact**
CI	NR	5.88 ± 4.88	−4.39 ± 3.64	−2.90 ± 2.43	154.00 ± 21.47	282.15 ± 36.32	38.08 ± 25.68
	LMR	12.99 ± 9.33	−5.60 ± 10.31	−3.76 ± 3.46	138.62 ± 23.38	267.54 ± 58.74	33.69 ± 21.82
	MAR	6.87 ± 3.26	−1.84 ± 4.09	−2.03 ± 2.63	138.08 ± 35.74	255.77 ± 72.23	25.77 ± 26.93
	REST	8.05 ± 4.82	−3.62 ± 5.48	−2.52 ± 3.21	133.38 ± 33.84	266.23 ± 56.95	25.0 ± 27.08
Control	NR	2.69 ± 3.43	−5.90 ± 5.74	−1.57 ± 2.31	132.80 ± 36.45	244.20 ± 71.96	23.93 ± 23.83
	LMR	4.12 ± 7.83	−12.72 ± 5.28	−2.95 ± 3.49	110.73 ± 22.67	270.73 ± 43.30	37.73 ± 29.10
	MAR	1.90 ± 3.54	−6.03 ± 2.76	−1.67 ± 1.57	108.40 ± 24.01	258.53 ± 57.08	32.27 ± 30.62
	REST	2.55 ± 4.74	−8.03 ± 3.50	−1.78 ± 2.24	113.00 ± 26.16	258.40 ± 56.84	33.67 ± 30.32

**Table 3:** Comparison of the CI group and control group on the level of different references.

**AEP component**	**Reference**	**Ability to distinguish the difference between CI and control group**
P1 A	NR	N, *p* = 0.135
	MR	Y, *p* = 0.000
	MAR	Y, *p* = 0.021
	REST	Y, *p* = 0.011
P1 L	NR	N, *p* = 0.052
	MR	Y, *p* = 0.011
	MAR	Y, *p* = 0.007
	REST	N, *p* = 0.062
N1 A	NR	N, *p* = 0.470
	MR	Y, *p* = 0.001
	MAR	Y, *p* = 0.046
	REST	Y, *p* = 0.036

**Table 4:** Comparison of different references in different groups.

	**CI**			**Control**		
**AEP component**	**Reference (I)**	**Reference (II)**	**Sig**	**Reference (I)**	**Reference (II)**	**Sig**
P1 A	NR	LMR	0.009^*^	NR	LMR	0.981
	NR	MAR	0.998	NR	MAR	0.999
	NR	REST	0.905	NR	REST	1.000
	LMR	MAR	0.037^*^	LMR	MAR	0.861
	LMR	REST	0.147	LMR	REST	0.971
	MAR	REST	0.995	MAR	REST	1.000
P1 L	NR	LMR	0.678	NR	LMR	0.200
	NR	MAR	0.643	NR	MAR	0.120
	NR	REST	0.346	NR	REST	0.310
	LMR	MAR	1.000	LMR	MAR	1.000
	LMR	REST	0.998	LMR	REST	1.000
	MAR	REST	0.999	MAR	REST	0.998
N1 A	NR	LMR	0.994	NR	LMR	0.006^*^
	NR	MAR	0.803	NR	MAR	1.000
	NR	REST	1.000	NR	REST	0.872
	LMR	MAR	0.406	LMR	MAR	0.007^*^
	LMR	REST	0.931	LMR	REST	0.120
	MAR	REST	0.957	MAR	REST	0.902

* *Significant difference exists (p < 0.05)*.

## P1 component

### P1 amplitude

Significant main effects of reference (*F* = 8.926, *p* = 0.001, adjusted by Greenhouse-Geisser) and group (*F* = 10.102, *p* = 0.004) with no reference^*^ group interaction (*F* = 3.139, *p* = 0.059, adjusted by Greenhouse-Geisser) were found on the P1 amplitude.

Further simple effect analysis showed that in the cochlear group, the amplitudes (mean = 12.99 μV, std = 9.33) using LMR were found to be significantly larger than that using NR (mean = 5.88 μV, std = 4.88) and MAR (mean = 6.87 μV, std = 3.26) (LMR&NR: *p* = 0.009; LMR&MAR: *p* = 0.037). No significant difference was found in amplitudes using these four references in the control group.

Using NR as a reference probe, no significant difference was found between the cochlear and control group for P1 amplitude (*p* = 0.135). While using LMR, REST or MAR as a reference, the difference among these groups reached a significant level (LMR: *p* = 0.000; MAR: *p* = 0.021; REST: *p* = 0.011) and using LMR led to less of a chance to make a type I error (LMR: *p* = 0.000; MAR: *p* = 0.021; REST: *p* = 0.011). REST had the least chance to make a type I error, besides LMR.

### P1 latency

For P1 latency, significant main effects of reference (*F* = 7.830, *p* = 0.002, adjusted by Greenhouse-Geisser) and group (*F* = 7.731, *p* = 0.010) were found with no significant reference^*^ group interaction (*F* = 0.440, *p* = 0.628, adjusted by Greenhouse-Geisser).

After the simple effect test, we found that whether in the CI or control group, no significant difference existed among the four references.

A difference in the CI and control group can be observed in LMR and MAR. In addition, MAR had less of a chance to make a type I error (LMR, *p* = 0.011; MAR, *p* = 0.007) in distinguishing the difference between the CI and control group.

## N1 component

### N1 amplitude

For N1 amplitude, significant main effects of reference (*F* = 7.999, *p* = 0.002, adjusted by Greenhouse-Geisser) and group (*F* = 7.604, *p* = 0.011) were found with no significant reference^*^ group interaction (*F* = 2.103, *p* = 0.139, adjusted by Greenhouse-Geisser).

With the result of the simple effect test, we acknowledged that in the CI group, there was no significant difference among the N1 amplitude the 4 references. However, in the control group, LMR (Mean = −12.72 μV, std = 5.28) showed a difference with NR (Mean = −5.90 μV, std = 5.74) and MAR (Mean = −6.03 μV, std = 2.76) (LMR&NR: *p* = 0.006; LMR&MAR: *p* = 0.007).

LMR, MAR, and REST could distinguish the difference of N1 amplitude between the CI and control group (LMR: *p* = 0.001; MAR: *p* = 0.046; REST: *p* = 0.036), while NR could not. In addition, LMR had the least chance to make a type I error.

### N1 latency

No significant main effects of reference (*F* = 0.412, *p* = 0.653, adjusted by Greenhouse-Geisser) or group (*F* = 0.325, *p* = 0.574) with no reference^*^ group interaction (*F* = 1.351, *p* = 0.213, adjusted by Greenhouse-Geisser) were found on N1 latency.

## Artifact

### Artifact amplitude

For artifact amplitude, no significant main effects in reference (*F* = 5.956, *p* = 0.007, adjusted by Greenhouse-Geisser) and group (*F* = 0.769, *p* = 0.389) were found. No reference^*^ group interaction (*F* = 0.549, *p* = 0.561, adjusted by Greenhouse-Geisser) existed.

### Artifact latency

For artifact latency, no significant main effects in reference (*F* = 1.095, *p* = 0.338, adjusted by Greenhouse-Geisser) or group (*F* = 0.020, *p* = 0.889) were found.

## Comparison of different differences

Contralateral MR has a greater ability in distinguishing CI children from the control group with less of a chance to make a type I error on P1 amplitude, while MAR did better on P1 latency. Further comparison can be found in the discussion.

NR and REST are not ideal for a P1 study, as they cannot distinguish two groups on P1 latency, which contrasts the present study.

## Discussion

In this study, the characteristics of the AEPs in three typical references were analyzed between the right-side-CI children and age-matched congenital left external and middle ear malformation children. Children used their implants for a similar time period (~1 year) on average. For P1, our results demonstrated that in the CI group, the amplitudes using LMR were found significantly larger than that using NR or MAR. However, no significant difference was found between amplitudes using NR and MAR. In addition, the REST result had no significant difference with the three other references. However, our results showed that different references for the AEP study did not affect the CI artifact. This might be due to that a 128 channel setup can help detect and reject CI artifacts (e.g., Artifact rejection) and replace the bad channels (e.g., bad channels replacement) (Luu et al., 2011).

Methodological differences between studies indicate that the chosen reference electrode location may determine which component is more prevalent in a given study. It is important to note and to insist on the fact that the topography of the potential field is completely independent of the choice of the reference (Geselowitz, 1998). Different references have been recommended for studies of different components (Wolpaw and Wood, 1982; Shih et al., 1988; Hagemann et al., 2001; Joyce and Rossion, 2005; Kulaichev, 2016). There has been some research adopting the nose as a reference because it is a long distance from the regions of interest, such as visual- and auditory-related regions (Banerjee et al., 2011; Tian and Yao, 2013). Duncan et al. (2009) reported that the preferred reference is the nose, as this method allows both frontal negative and mastoid positive aspects of the signal to be visualized and measured. In addition, Shafer et al. (2015) reported that studies using a mastoid or NR will show a relatively prominent Tb peak (compared to Na). The underlying principle of average reference is that the electrical events produce both positive and negative poles. The integral part of these potential fields in a conducting sphere sums to exactly zero (Bertrand et al., 1985; Dien, 1998). It is important to note that for CI users, implant devices create electrical artifacts on the scalp; these artifacts might lead to outlier potentials, which affect the average. For this reason, the average reference may not be suitable for all components of the AEP study. To reduce electrical artifacts, it is well accepted that contralateral mastoid as the reference electrode is one of the best references for AEPs of CI users (He et al., 2012; Mc Laughlin et al., 2012, 2013; Miller and Zhang, 2014). Meanwhile, our results showed that different references for the AEP study do not affect the CI artifact.

The P1 component originating from the primary auditory cortex and thalamus reflects the summed synaptic transmission along the ascending auditory pathway (Sharma et al., 2015b), which can assess the maturation of the central auditory system via changes in latency and amplitude (Ponton et al., 2000; Wunderlich and Cone-Wesson, 2006; Wunderlich et al., 2006; Shafer et al., 2015). In CI children who received an implant before age 3.5 years, the latency and amplitude of the P1 component of the AEPs decrease rapidly and finally reach the normal age range (Sharma et al., 2002; Kral and Sharma, 2012). Our findings suggest that latencies of the P1 peak were significantly longer, and amplitudes were significantly larger in the CI than in the control group, which are consistent with previous studies (Eggermont and Ponton, 2003). Cortical ERPs mainly reflect the postsynaptic activity in pyramidal neurons, which is subject to the largest spatial and temporal summation, with each pyramidal cell neuronal column behaving as an electrical dipole (Steinschneider et al., 2011). Thus, our studies indicate immaturity of the primary auditory cortex in CI children; the transmission and synaptic delays along peripheral and central auditory pathways became longer, and the synchronization of neurons became poor after periods of auditory deprivation. The AEPs of CI children exhibited broader neural firing and formed broader positive potentials and higher P1 amplitudes over the cortex.

Although the midline electrodes (e.g., Fz, Cz, Pz) were usually used in the ERP studies, it had been reported that the early components (i.e., P1–N1) of AEP had a significant higher distribution in fronto-central areas, and Fz electrode site is most frequently used in studying P1–N1 components for its obvious observation (Brandwein et al., 2011; He et al., 2012; Cooper et al., 2013). In addition, in our previous studies, we also found that the Fz was a suitable electrode site (easy to distinguish P1–N1) in an auditory ERP study in normal and CI children (Zheng et al., 2011; Liang et al., 2014). Furthermore, a 128 channel setup can help minimize the error during the EEG data analysis (e.g., Artifact rejection, Bad channels) (Luu et al., 2011). Therefore, only the Fz was chosen for the present study, though the 128-channel setup was used. However, for the reference electrode in our study, we found that the amplitudes using LMR were significantly larger than other references in the CI group. The positive potential on the Fz electrode may be neutralized while using NR and MAR reference, which leads to the decline of the P1 amplitude. As the ERP components suggest to be determined by subjective visual observation (Zheng et al., 2011), a more apparent P1 should be preferable for the clinical ERP test. Therefore, the selection of the contralateral MR can be suitable for the test of the P1 response on Fz.

The positive potential on the Fz electrode may be neutralized while using NR and MAR, which leads to the decline of the P1 amplitude. With a relatively inactive contralateral mastoid as the reference electrode, the spatial distance between the recording electrode and reference electrode increases; then, the P1 amplitude should be more prominent. In the control group whose children had moderate-severe conductive hearing loss in the left ear and normal hearing in the right ear, the auditory cortex would be much more mature, and the positive potential would then be more concentrated, which results in shorter latency and a smaller amplitude of the P1 component. The choice of reference makes little difference in the control group. However, our present study found that it was easier to enhance the amplitude differences between the CI and control group with a smaller variation while using LMR. Furthermore, the use of LMR achieved the minimal type I error. Therefore, we assumed that contralateral MR should be a more preferable reference in AEP studies, as it has a greater ability to distinguish CI children from children with ear malformation, while studying P1 amplitudes. In addition, we recommend contralateral MR as the reference to assess the mature degree of the P1 component in the CI group. In addition, we used a one side MR to reduce the interference of the implanted cochlear, which is contrary to the traditional two side MRs. MAR and MR are acceptable for the P1 and N1 study. In addition, REST is acceptable only for N1 studies. Generally, taking the more obvious P1 amplitude into consideration, contralateral MR is more ideal for the N1–P1 component study.

## Conclusion

P1 amplitude is significantly larger with contralateral MR than with NR and MAR and has a greater ability to distinguish CI children from children with ear malformation, with less of a chance to make a type I error. MAR and MR can distinguish the difference of two groups on P1 latency, and MAR is less likely to make type I errors. We recommend contralateral MR or MAR as an acceptable reference in the AEP P1 component study in CI patients. Considering that MR also showed greater P1 amplitude, contralateral MR is a more ideal choice for a general AEP study. REST is acceptable to study the N1 component.

NR is not acceptable for P1 or N1 studies. Different references for AEP studies do not affect the CI artifact.

## Author contributions

ML: Designed the experiment, interpreted the results, and wrote the manuscript; JL: Performed the experiment, analyzed the data, and wrote the manuscript; JZ: Interpreted the results and revised the manuscript; JW: Analyzed the data; YCh: Helped to improve the experiment and the paper; YCa: Did some work on discussion; LC: Helped to collect patient; YZ: Supervised the work. All authors read and approved the final manuscript.

### Conflict of interest statement

The authors declare that the research was conducted in the absence of any commercial or financial relationships that could be construed as a potential conflict of interest.
